# Supplementary material for: A Critical View of the Application of the APEX Software (Aqueous Photochemistry of Environmentally-Occurring Xenobiotics) to Predict Photoreaction Kinetics in Surface Freshwaters
Source: Molecules. 2019 Dec 18;25(1):9. doi: 10.3390/molecules25010009 (PMC7017383; doi:10.3390/molecules25010009)
Supplement: Supplementary file 1 [file molecules-25-00009-s001.zip › 3.2.4_gcc-4.4.0.zip!/3.2.4_gcc-4.4.0/3.2.4_gcc-4.4.0/doc/octave/html/interpreter/Cell-Arrays-with-Mex_002dFiles.html]

Cell Arrays with Mex-Files - Untitled


Next: Structures with Mex-Files,
Previous: Character Strings in Mex-Files,
Up: Mex-Files


---

#### A.2.4 Cell Arrays with Mex-Files

We can perform exactly the same operations in Cell arrays in mex-files
as we can in oct-files. An example that reduplicates the functional of
the celldemo.cc oct-file in a mex-file is given by
mycell.c as below

```
```
     #include "mex.h"
     
     void
     mexFunction (int nlhs, mxArray* plhs[], int nrhs, 
     	     const mxArray* prhs[])
     {
       mwSize n;
       mwIndex i;
     
       if (nrhs != 1 || ! mxIsCell (prhs[0]))
         mexErrMsgTxt ("expects cell");
     
       n = mxGetNumberOfElements (prhs[0]);
       n = (n > nlhs ? nlhs : n);
       
       for (i = 0; i < n; i++)
         plhs[i] = mxDuplicateArray (mxGetCell (prhs[0], i));
     }
```
```

which as can be seen below has exactly the same behavior as the oct-file
version.

```
     [b1, b2, b3] = mycell ({1, [1, 2], "test"})
     =>
     b1 =  1
     b2 =
     
        1   2
     
     b3 = test
```

Note in the example the use of the `mxDuplicateArray` function. This
is needed as the `mxArray` pointer returned by `mxGetCell`
might be deallocated. The inverse function to `mxGetCell` is
`mcSetCell` and is defined as

```
     void mxSetCell (mxArray *ptr, int idx, mxArray *val);
```

Finally, to create a cell array or matrix, the appropriate functions are

```
     mxArray *mxCreateCellArray (int ndims, const int *dims);
     mxArray *mxCreateCellMatrix (int m, int n);
```
